# Development and Validation of a Three-Gene Prognostic Signature Based on Tumor Microenvironment for Gastric Cancer

**DOI:** 10.3389/fgene.2021.801240

**Published:** 2022-02-01

**Authors:** Qian Wang, Xiangmei Li, Yahui Wang, Jiayue Qiu, Jiashuo Wu, Yalan He, Ji Li, Qingfei Kong, Junwei Han, Ying Jiang

**Affiliations:** ^1^ College of Bioinformatics Science and Technology, Harbin Medical University, Harbin, China; ^2^ College of Basic Medical Science, Harbin Medical University, Harbin, China; ^3^ College of Basic Medical Science, Heilongjiang University of Chinese Medicine, Harbin, China

**Keywords:** gastric cancer, prognostic signature, tumor microenvironment, survival analysis, three-gene prognostic signature

## Abstract

Gastric cancer (GC), which has high morbidity and low survival rate, is one of the most common malignant tumors in the world. The increasing evidences show that the tumor microenvironment (TME) is related to the occurrence and progression of tumors and the prognosis of patients. In this study, we aimed to develop a TME-based prognostic signature for GC. We first identified the differentially expressed genes (DEGs) related to the TME using the Wilcoxon rank-sum test in a training set of GC. Univariate Cox regression analysis was used to identify prognostic-related DEGs. To decrease the overfitting, we performed the least absolute shrinkage and selection operator (LASSO) regression to reduce the number of signature genes and obtained three genes (LPPR4, ADAM12, NOX4). Next, the multivariate Cox regression was performed to construct the risk score model, and a three-gene prognostic signature was developed. According to the signature, patients were classified into high-risk and low-risk groups with significantly different survival. The signature was then applied to three independent validated sets and obtained the same results. We conducted the time-dependent Receiver Operating Characteristic (ROC) curve analysis to evaluate our signature. We further evaluated the differential immune characters between high-risk and low-risk patients to reveal the potential immune mechanism of the impact on the prognosis of the model. Overall, we identified a three-gene prognostic signature based on TME to predict the prognosis of patients with GC and facilitate the development of a precise treatment strategy.

## Introduction

Gastric carcinoma (GC) is one of the third leading causes of cancer-related deaths in the world, which has high morbidity and mortality (Morbidity = 5.7%; Mortality = 8.2%) (Bray et al., 2018). The death rate of gastric cancer has indeed decreased, but gastric cancer is still one of the major diseases endangering human life (Siegel et al., 2021). Although many studies have explored the pathogenesis of the GC, it remains to be further confirmed (KankeuFonkoua and Yee, 2018). A large number of studies have suggested that TME is related to tumor progression and patient survival outcomes in recent years (Quail and Joyce, 2013). More and more evidence shows that TME has clinic pathological significance in predicting the effect of treatment (Binnewies et al., 2018; Zeng et al., 2019). Therefore, it is essential to develop a TME-based model to predict the prognosis of patients with GC and guide a more effective treatment strategy.

The TME is a complex tissue that consists of tumors, mesenchymal stem cells, fibroblasts, endothelial cells, inflammatory cells, and extracellular matrix. As the main components of TME, immune infiltrating cells and stromal cells have attracted more and more attention. The evaluation of the status of these two types of cells in TME will help to more accurately evaluate the prognosis of tumor patients. Nowadays, many bioinformatics tools can be used to evaluate the distribution of immune cells and stromal cells in TME. Among them, the Estimation of STromal and Immune cells in MAlignant (ESTIMATE) is widely applied for the quantitative analysis of TME (Yoshihara et al., 2013). It can infer the infiltration of stromal and immune cells using gene expression signatures. This algorithm has been employed in glioblastoma (Jia et al., 2018), cell renal cell carcinoma (Xu et al., 2019), and colon cancer (Alonso et al., 2017) successfully in recent years. However, there is no detailed analysis of the immune, extracellular matrix, and estimate scores of the GC.

The study of cancer prognosis plays an important role in cancer research. With the development of high-throughput sequencing, several risk-stratification models which can predict the prognosis of patients with GC have been produced, but few of them were useful in clinical practice. At present, the prognosis of the GC patients mainly depends on clinic pathological features, such as tumor histology, stage, and grade. However, the existing conventional methods are insufficient to meet the current rapidly increasing clinical demand. Therefore, we established a risk score model based on gene expression data and the list of genes related to the TME for predicting the prognosis of the GC patients.

In this study, we conducted a differential expression analysis of the gene signatures related to the TME. The Cox regression analysis and LASSO regression analysis were performed on further screening gene signatures related to the prognosis of patients with GC. We then constructed a risk score model and identified a TME-based three-gene prognostic signature with the ability to predict the overall survival of patients with GC. The signature prognostic value was validated in three independent data sets. Finally, we applied the time-dependent ROC curve analysis to verify our signature and analysis the immune status between high-risk and low-risk groups to reveal the potential immune mechanism of the impact on the prognosis of the model.

## Materials and Methods

**FIGURE 1:**
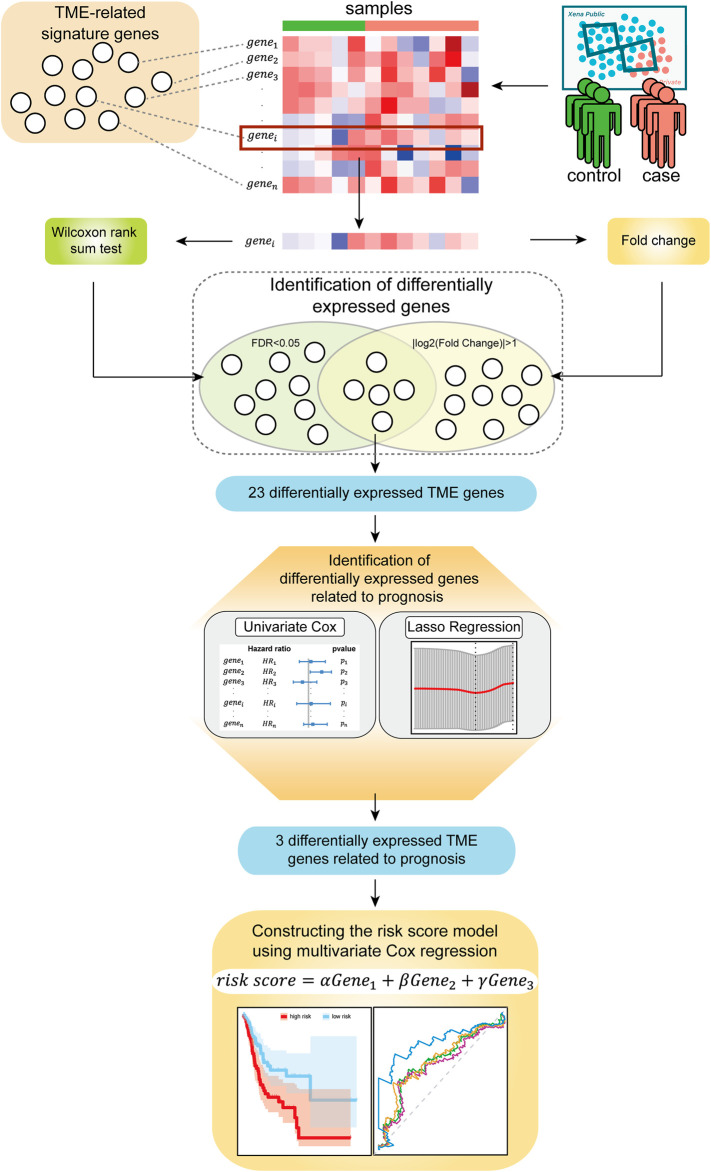
Flow diagram of the study.

In this study, we explored biomarkers related to the TME of the GC based on mRNA expression profiles via bioinformatics approaches. We describe the flow of this study in Figure 1. This study mainly consists of five parts: (Bray et al., 2018) the mRNA expression profile of the GC samples and the signature genes related to the TME were sorted into a gene expression profile, which was related to the immune microenvironment of GC; (Siegel et al., 2021) genes that were significantly differentially expressed between normal samples and disease samples were identified using the Wilcoxon rank-sum tests and Fold Change; (KankeuFonkoua and Yee, 2018) the univariate Cox regression analysis and LASSO regression analysis were used to further screen prognostic-related signature genes; (Quail and Joyce, 2013) the three identified signature genes were incorporated into the multivariate Cox regression analysis for constructing risk score model; (Zeng et al., 2019) the samples were divided into a high-risk group and a low-risk group based on the risk score model and then the prognostic ability and robustness of the risk score model were evaluated.

### Datasets

Fragments per Kilobase Million (FPKM) normalized expression profile data of the patients for Stomach Cancer (STAD) of GDC TCGA were downloaded from the UCSC Xena database (https://xena.ucsc.edu/). And then the clinical information of the GC patients was also downloaded from the database and combined into a single file for further analysis. A total of 407 samples were obtained. Three independently validate data sets containing microarray expression data and related clinical information of the GC patients, GSE26901, GSE13861, and GSE15459, were collected from the GEO database (https://www.ncbi.nlm.nih.gov/geo/). Detailed information and sources of the above datasets used in this study are listed in Supplementary Table S1. For each dataset, the average expression value was calculated when the same gene corresponds to multiple probes. And we performed z-score standardization on the three independent validation sets.

### Identification of Differentially Expressed Genes Related to GC Tumor Microenvironment

ESTIMATE is an algorithm to infer the level of immune cell and stromal cell infiltration in tumor tissues based on transcriptome data of TME-related genes containing a set of immune and stromal signature genes (Yoshihara et al., 2013). The algorithm contains 282 feature genes related to the TME, including 141 immune feature genes and 141 stromal feature genes. Here we studied the expression profile of TCGA-STAD containing 282 feature genes related to the TME. After removing the genes whose expression value is 0 in all samples, we obtained an expression profile containing 280 feature genes related to the TME. We identified TME-related genes that are significantly differentially expressed between normal samples and tumor samples using the Wilcoxon rank-sum test, and controlled the False Discovery Rate (FDR) in multiple comparisons by the Benjamini-Hochberg procedure (Benjamini and Hochberg, 1995). The screening criteria were |log2(Fold Change)| > 1 and FDR < 0.05.

To explore the main biological functions of differentially expressed genes related to the TME, we performed Gene Ontology (GO) enrichment analysis on the differentially expressed genes using the clusterProfiler package (Yu et al., 2012). The GO terms with FDR < 0.05 were statistically significant enrichment analysis results.

### Construction of the Risk Score Model and Evaluation of its Prediction Performance

We performed z-score standardization on gene expression data for the next steps. The univariate Cox regression analysis was performed on 333 samples with both gene expression and survival data to determine the relationship between differentially expressed TME genes and the overall survival (OS), and screened genes with *p* < 0.05. Then, LASSO regression analysis was further performed to identify signature genes by the glmnet package in R software. LASSO regression is a method commonly applied for fitting selecting variables in high-dimensional generalized linear models (Friedman et al., 2010). It obtains a more refined model by constructing a penalty function, effectively avoiding overfitting. Recently, the LASSO regression has been expanded to the Cox proportional hazard regression model for survival analysis (Tibshirani, 1997). With the identified signature genes from LASSO regression, we finally constructed a risk score model by using the multivariate Cox regression analysis. The formula for the risk score is given as:

$$Riskscore=\sum_{k\in S}\beta_{k}{\text{α}}_{\text{k}}$$ (1)

where *S* is a set of the signature genes obtained from the LASSO regression; *a*_*k*_ is the expression level of signature genes *k; β*_*k*_ is the regression coefficient of the multivariate Cox proportional hazard regression model estimated on *a*_*k*_ and the overall survival data.

According to the risk score model, the risk score of each sample is calculated, and the samples were divided into a high-risk group and a low-risk group based on the median risk score. Kaplan-Meier (KM) survival analysis and time-dependent receiver operating characteristic (ROC) curve analysis were used to evaluate the predictive performance of the risk score model. The prognostic effects of other clinicopathologic factors were also evaluated.

### Evaluation of the Correlations of Immune Characters and Risk Score

To reveal the potential immune mechanism of the impact on the prognosis of the model, we evaluated the correlations of TME-related characters and risk scores. The infiltration levels of immune cells were inferred based on the feature genes of cells with the single-sample gene-set enrichment analysis (ssGSEA) method, which was implemented by the SMDIC package developed by our group (https://CRAN.R-project.org/package=SMDIC) (Jiang et al., 2021). The Wilcoxon rank-sum test was used to test the differential infiltrated levels of cells between the high-risk group and low-risk group. Then, the ESTIMATE algorithm was applied to calculate the immune score, stromal score, ESTIMATE score, and tumor purity of the GC patients. We explored the correlation between these four features and the risk score using the linear fitting method.

Immune checkpoints are a class of genes that suppress immune effects. They are expressed on immune cells to regulate the degree of immune activation and prevent autoimmune effects. The role of immunotherapy is to overcome the immune suppression caused by the tumor and its microenvironment so that the immune system can play a normal role and target cancer cells (Billan et al., 2020). Abnormal expression and function of immune checkpoint genes can cause many diseases. For example, overexpression or over-functioning of immune checkpoint molecules can inhibit immune function, reduce the body’s immunity, and prone to diseases such as tumors. Therefore, to further learn the potential immune mechanism of the impact on the prognosis of the model, we assessed the differences in the gene expression of the three immune checkpoints: programmed cell death 1 (PD-1) or ligand 1 (PD-L1), and cytotoxic T-lymphocyte antigen-4 (CTLA-4) between the high-risk group and the low-risk group.

Moreover, Human Leukocyte Antigen (HLA) is a protein molecule that exists on the surface of antigen-presenting cells and is responsible for antigen presentation. It is a sign that different individual immune cells recognize each other, participates in the immune response, and have very important biological functions (Dendrou et al., 2018). We also analyzed the differences in HLA gene expression between the high-risk group and low-risk group based on gene expression data.

### Functional Enrichment Analysis of the Signature Genes

We then detected the biological function of the signature genes in the risk score model. For each signature gene, we classified the samples into two classes according to the median of gene expression level, and the GSEA method was applied to identify the pathways affected when the expression level of the signature gene changes to explore the biological pathways affected by the gene (Subramanian et al., 2005). The KEGG pathways were used and were downloaded from the MSigDB database (https://www.gsea-msigdb.org/gsea/msigdb/) (Subramanian et al., 2005).

## Results

### Identification of Differential Expression Genes Related to TME in GC

**FIGURE 2:**
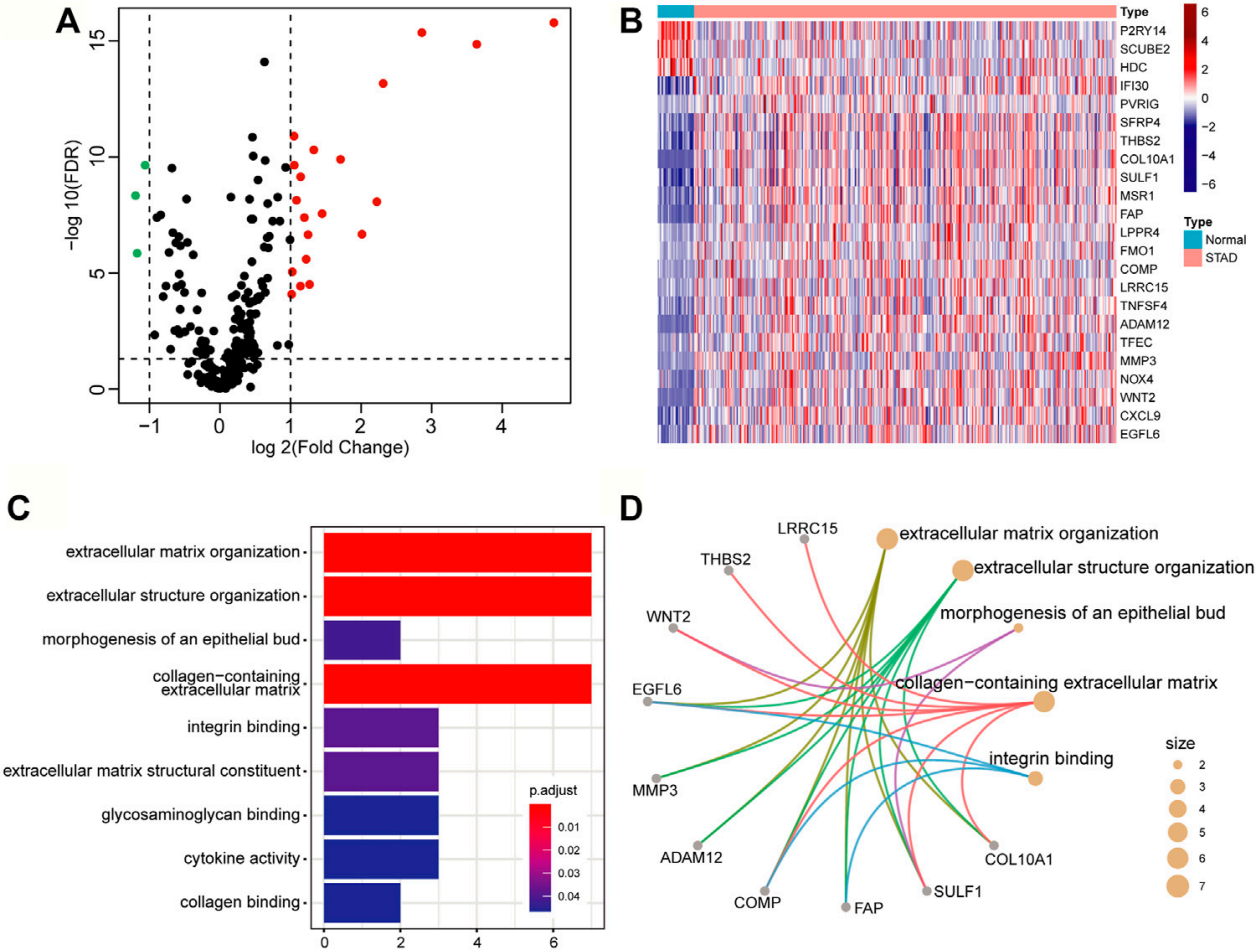
Differentially expressed genes related to the tumor microenvironment. **(A)** The volcano map of the TME-related differential expressed genes. The screening criteria were |log2(Fold Change)| > 1 and FDR < 0.05. The red, green, and black points represent genes that were significantly up-regulated, down-regulated, and insignificant. **(B)** Heatmap of significantly TME-related differentially expressed genes. **(C)** Bar graph of GO enrichment analysis of significantly TME-related differentially expressed genes. **(D)** The main terms and related genes for GO enrichment analysis. The size of the circle corresponds to the number of genes annotated to the terms.

A number of studies have shown that the occurrence and development of tumors and the prognosis of patients are related to the TME. We intend to develop a TME-based gene prognostic signature for GC. To do this, we collected 282 TME-related signature genes (141 immune feature genes and 141 stromal feature genes) from ESTIMATE (Yoshihara et al., 2013), which were annotated to gene expression data of STAD in TCGA. The Wilcoxon rank-sum test and Fold Change analysis were used to identify the TME-related differentially expressed genes between normal samples and tumor samples. With FDR < 0.05 and |log2(Fold Change)| > 1, 23 differentially expressed genes (20 significantly up-regulated genes and 3 significantly down-regulated genes) were identified (Figure 2A). As shown in the heatmap of these 23 differentially expressed genes, they are classified into two distinct blocks between normal and tumor samples (Figure 2B).

To insight into the biological function of the differentially expressed genes, we performed GO enrichment analysis by using the clusterProfiler package. With FDR<0.05, these differential expression genes were mainly annotated in nine GO terms, such as extracellular matrix organization (GO:0030198), extracellular structure organization (GO:0043062), and collagen-containing extracellular matrix (GO:0062023), etc. (Figure 2C). Several genes, such as EGFL6 (EGF Like Domain Multiple 6), COMP (Cartilage Oligomeric Matrix Protein), and SULF1 (Sulfatase 1), have been found to be related to multiple terms (Figure 2D), and they are all research-proven cancer-related biomarkers. For example, EGFL6 and COMP are highly associated with colon cancer (Cao et al., 2018; Nfonsam et al., 2020). SULF1 can promote drug-induced apoptosis of cancer cells *in vitro*, and inhibit tumorigenesis and angiogenesis *in vivo* (Lai et al., 2008).

### Construction and Verification of a TME-Based Three-Gene Prognostic Signature

**FIGURE 3:**
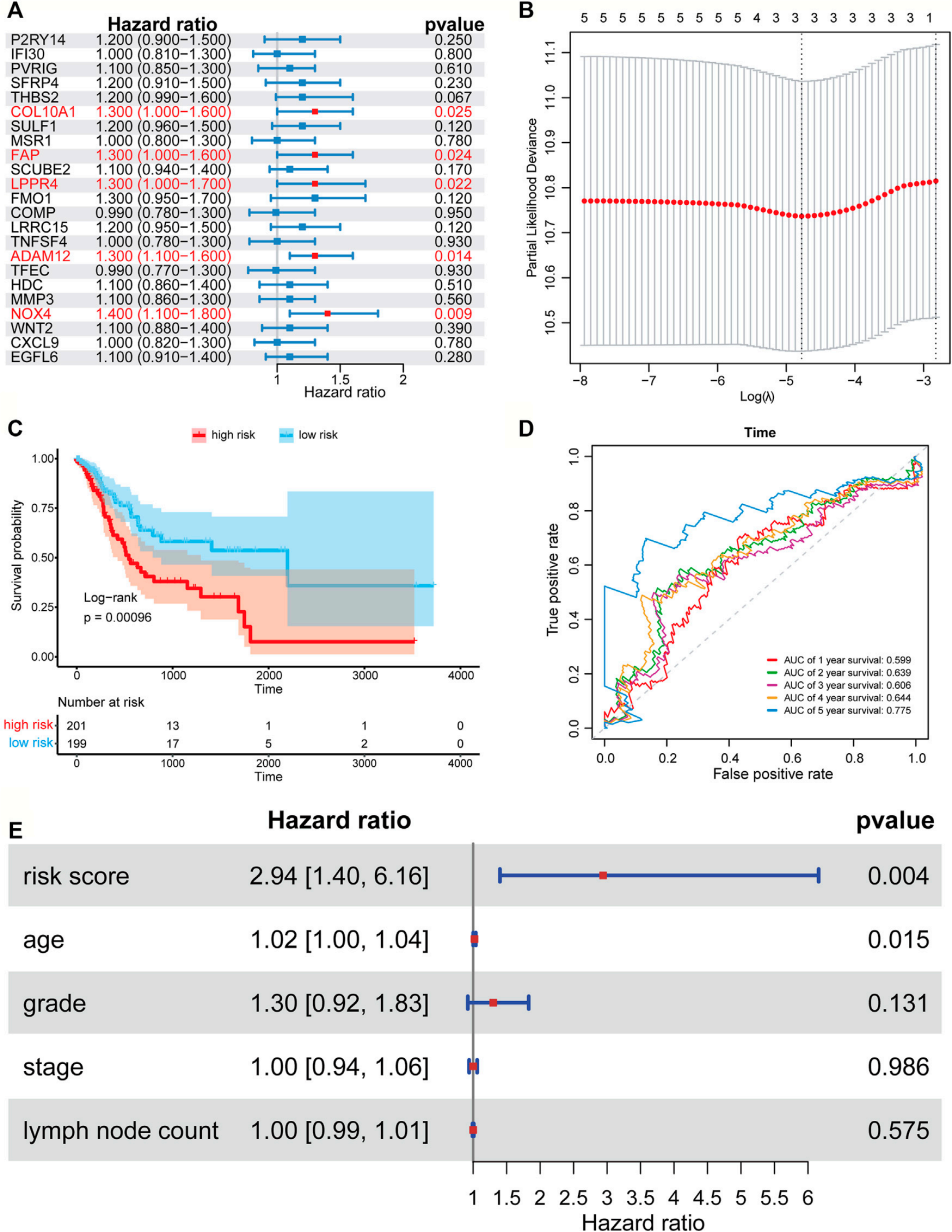
Forest plots, Kaplan-Meier plots, and time-dependent ROC curves of the risk score model. **(A)** Forest plots of univariate Cox proportional hazards regression analysis identified five prognostic genes related to the TME. **(B)** LASSO regression analysis showed the partial likelihood deviation curve of the minimum number genes corresponding to the covariates used for multivariate Cox analysis. **(C)** The Kaplan-Meier curves of the high-risk and low-risk group. **(D)** ROC curves chart of the risk score model predicting the 1–5 years survival rate. **(E)** Multivariate Cox regression analysis of risk score and other prognostic factors.

We applied the univariate Cox proportional hazard regression to the 23 TME related differentially expressed genes, and five statistically significant genes were identified (*p* < 0.05) (Figure 3A). Then, we applied LASSO regression analysis to the five genes to further identify the minimal feature genes. Three feature genes, LPPR4 (Lipid Phosphate Phosphatase-Related Protein Type 4), ADAM12 (ADAM metallopeptidase domain 12), and NOX4 (NADPH Oxidase 4), were obtained to constructing a prognostic signature (Figure 3B). Finally, we derived a formula to calculate the risk score of the signature for every patient from the expression values of the three genes weighted by the multivariate Cox proportional hazard regression coefficient (Supplementary Table S2):

riskscore=0.205×LPPR4 expression+0.146×ADAM12 expression+0.131×NOX4 expression (2)

According to the median risk score (0.403), a patient in the TCGA-STAD data set was classified as high risk if the risk score was higher than the median risk score and as low risk if was not. The Kaplan-Meier curve and time-dependent ROC curve were employed to evaluate the effect of the risk model for the three-gene prognostic signature in predicting the survival outcome of patients with GC. The overall survival (OS) difference between the high-risk group and low-risk group classified by the risk score model was showed to be significant in the TCGA training set (log-rank tests, *p* = 0.00096) (Figure 3C). The area under the ROC curve (AUC) for predicting 1–5 years survival rate was 0.605, 0.636, 0.602, 0.639, and 0.779 (Figure 3D), which suggests that the three-gene prognostic signature could predict the patient survival of GC. To test if our risk score is an independent prognostic factor, we further performed multivariate Cox proportional hazard regression to analyze the prognostic effects of the risk score and other common clinical factors, including age, grade, stage, and lymph node count (Figure 3E). The results showed that the three-gene signature could be used as an independent prognostic factor to predict the overall survival of GC patients.

**FIGURE 4:**
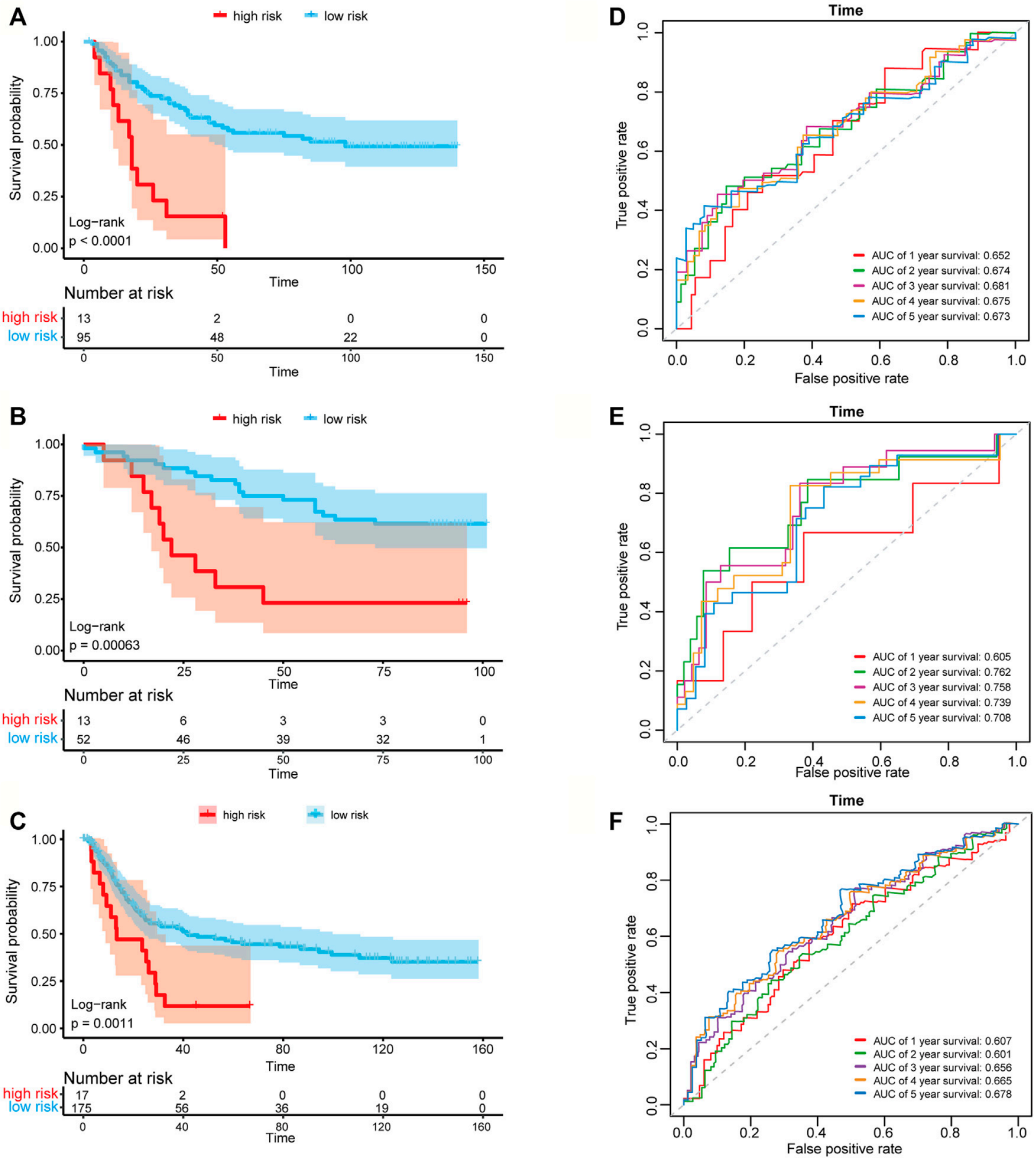
Kaplan-Meier plots and time-dependent ROC curves of the risk score model in three GEO data sets. Kaplan-Meier survival curve shows the overall survival of high-risk and low-risk groups in **(A)** GSE26901 data set, **(B)** GSE13861 data set and **(C)** GSE15459 data set. ROC curve plots for predicting the 1–5 years survival rate in **(D)** GSE26901 data set and **(E)** GSE13861 data set and **(F)** GSE15459 data set.

We then used the risk score of the three-gene signature to classify patients from the three independent GC data sets (GSE26901, GSE13861, and GSE15459) to validate whether the three-gene signature had the same or similar prognostic value in different populations. Consistent with the results in the training set, the signature could divide the samples of the GEO data sets into high-risk and low-risk groups, and the overall survival between the samples in the high-risk and low-risk groups in the three data sets showed to be a significant difference (log-rank tests, *p*-value in GSE26901 < 0.001, *p*-value in GSE13861 = 0.00063, and *p*-value in GSE15459 = 0.0011) (Figures 4A–C). The AUC values in GSE26901 data set for 1–5 years were 0.652, 0.674, 0.681, 0.675, and 0.673 (Figure 4D). Similar results were obtained in the GSE13861 and GSE15459 data sets (Figures 4E,F). The above analysis proved that the three-gene signature has high accuracy and robustness in predicting the overall survival of patients with GC.

### Functional Enrichment Analysis of the Three Signature Genes

The three genes (LPPR4, ADAM12, and NOX4) in the prognostic signature have been reported to be associated with GC. For example, the expression of LPPR4 was found to be increased in peritoneal metastasis of GC tissues, and high LPPR4 expression was associated with poor overall survival in GC (Zang et al., 2020). ADAM12 is highly expressed in cancer of gastric and is implicated in the malignant growth of GC cells (Carl-McGrath et al., 2005). NOX4 was found to be a new genetic target for anti-cancer therapy in digestive system cancer such as GC (Tang et al., 2018). Pathways are models containing the interaction, regulation, modification, and binding, etc. between genes, which could be used to dictate disease states, tumor marker, drug response, and altered cellular function (Di et al., 2019; Liu et al., 2019; Han et al., 2020; Han et al., 2021; Sheng et al., 2021).

**FIGURE 5:**
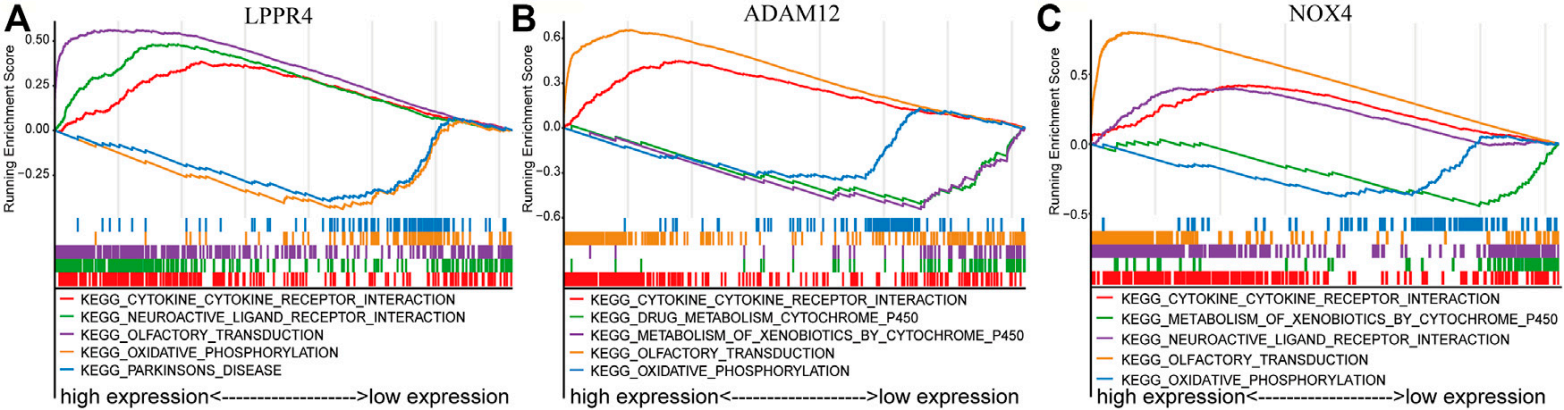
GSEA enrichment analysis of three signature genes related to TME. The top five most significant pathways were shown for each gene. **(A)** LPPR4. **(B)** ADAM12. **(C)** NOX4.

To further explore the potential mechanisms of the prognostic effects of the three signature genes, we performed GSEA to identify abnormal pathways for each gene in the TCGA data set. Specifically, for each gene, we classified the samples into two classes (high-risk and low-risk groups) according to the median of gene expression value, and the pathways were annotated to the differential expression gene list between the two classes (see Method). We presented the top five most significant pathways for each gene (Supplementary Table S3). The results showed that the abnormal pathways regulated by the three genes are quite consistent (Figures 5A–C). Specifically, the high expression of the three genes was all associated with the cytokine-cytokine receptor interaction pathway. The cytokines were generally produced by stimulated cells, mainly immune cells (Zhang and An, 2007), which suggested that the prognosis of the GC patients was related to immune activity. The low expression of these genes was associated with the oxidative phosphorylation pathway. Inhibition of oxidative phosphorylation has an impact on the intrinsic antitumor immune response (Boreel et al., 2021). Moreover, the lowly expressed of ADAM12 and NOX4 was associated with the metabolism of xenobiotics by the cytochrome P450 pathway. The cytochrome P450 were proposed to be key enzymes in cancer formation and cancer treatment (Rodriguez-Antona and Ingelman-Sundberg, 2006). These results indicated that the three genes of the prognostic signature are associated with the process of cancer and immune response.

### Evaluation of Differential Immune-Related Cells and Genes Between High-Risk and Low-Risk Groups

**FIGURE 6:**
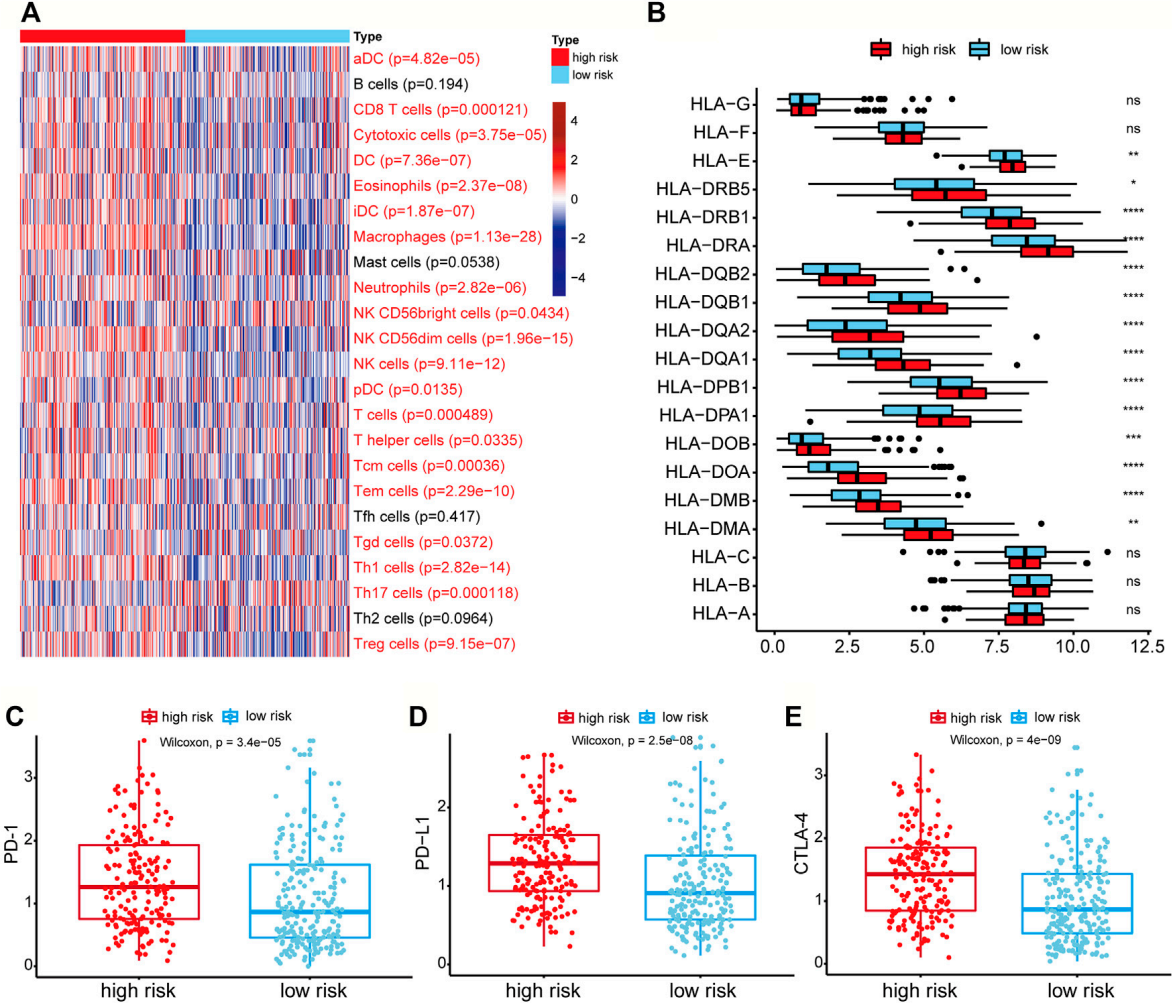
Differential immune-related cells and genes between the high-risk group and low-risk group. **(A)** Heatmap of the activities of 24 immune cells between high-risk and low-risk groups analyzed by ssGSEA. The *p*-value was calculated by the Wilcoxon rank-sum test. Immune cells with red labels referred to *p* < 0.05. **(B)** The expression of HLA-related genes between high-risk and low-risk groups. ns: *p* > 0.05, *p < 0.05, **p < 0.01, ***p < 0.001, ****p < 0.0001. **(C–E)** Genes expression of the PD-1, PD-L1, CTLA4 in the high-risk and low-risk groups.

The 24 immune cell type-specific gene signatures were obtained from the Bindea et al. publication (Bindea et al., 2013), and the ssGSEA method was applied to infer the relative tumor infiltration levels of the 24 immune cells. This process was implemented with our SMDIC package (https://CRAN.R-project.org/package=SMDIC) (Jiang et al., 2021). The Wilcoxon rank-sum test was used to identify the differentially infiltrated immune cells between high-risk and low-risk groups. 20 of 24 cells showed a significant difference (*p* < 0.05), and most of which were upregulated in the high-risk groups (Figure 6A). For example, the infiltration level of CD8^+^ T cell was higher in the high-risk group than in the low-risk group. It has been reported that tumors with either high CD8^+^ T cell density had worse overall survival (Thompson et al., 2016).

As the HLA-related genes play a key role in the induction and regulation of immune responses (Mosaad, 2015), we then evaluated the difference of HLA-related genes between high-risk and low-risk groups. The results showed that most HLA-related genes were expressed higher in the high-risk group (Figure 6B). It indicated that the patients in higher-risk group had higher immune cell activity and poor prognosis, which may be regulated by the high expression of HLA-related genes. We further evaluated the differential expression levels of three immune checkpoint genes (PD-1, PD-L1, and CTLA-4) between high-risk and low-risk groups to explore the relationship between immune checkpoint genes and risk score. We found that the expression levels of PD-1, PD-L1, and CTLA-4 in the high-risk group were all higher than those in the low-risk group (Wilcoxon rank-sum test, *p*-value of PD-1 = 3.4e-05, *p*-value of PD-L1 = 2.5e-08, and *p*-value of CTLA-4 = 4e-09) (Figures 6C–E). These results showed that the high expression of immune checkpoint genes correlated with the poor prognosis of patients, and patients with high expression of immune checkpoints might be more suitable for corresponding immunotherapy.

### Evaluation of the Correlation of Risk Score With the Immune Score, Stromal Score, and Tumor Purity

**FIGURE 7:**
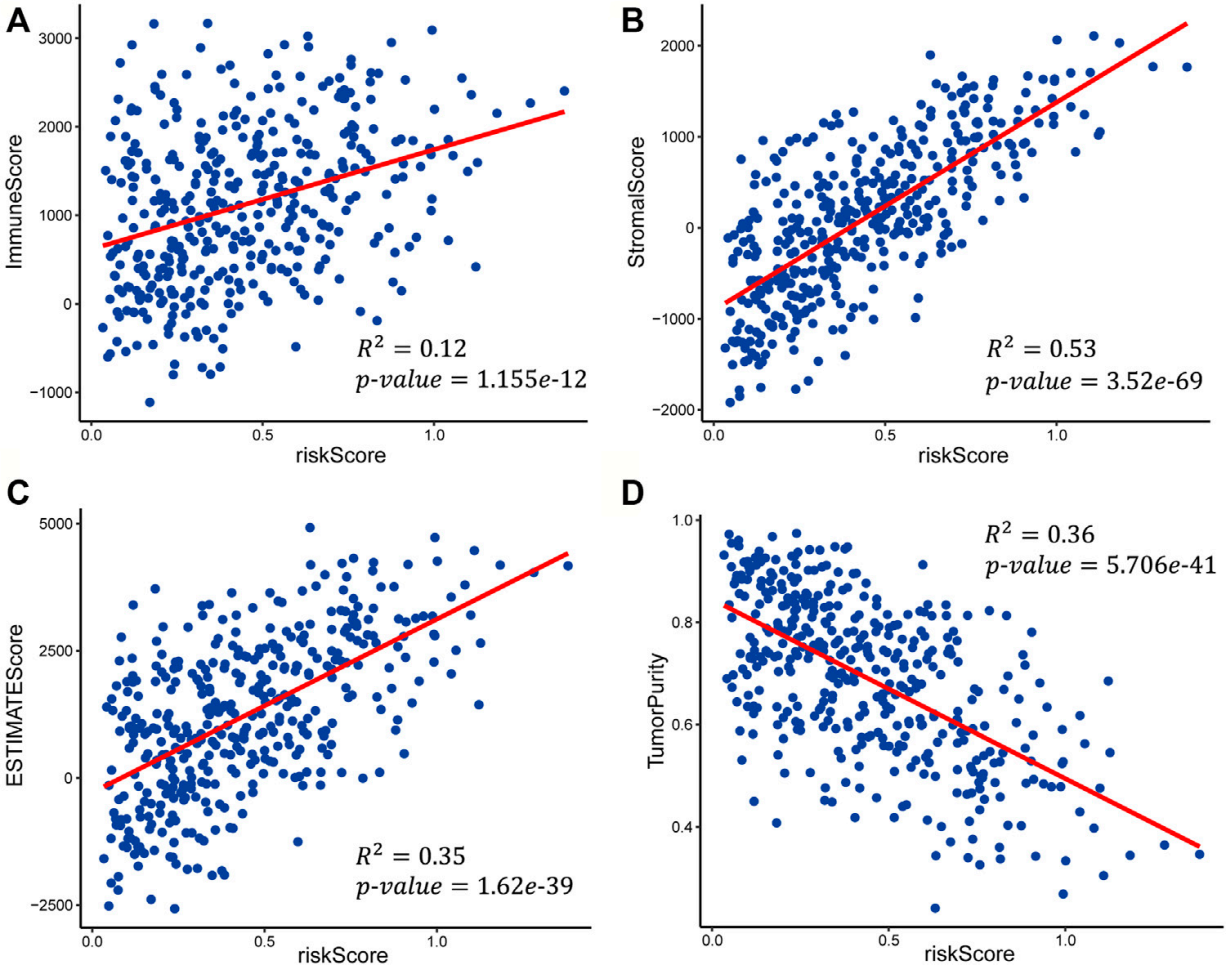
Scatter plots of the linear fit of risk score with **(A)** immune score, **(B)** stromal score, **(C)** ESTIMATE score, and **(D)** tumor purity.

We calculated the immune score, stromal score, ESTIMATE score, and tumor purity of the high-risk and low-risk groups with the ESTIMATE algorithm to explore the correlation between the patient’s immune activity and risk score. Our results indicated that the four scores showed significant differences between the high-risk and low-risk groups. The immune score, stromal score, and tumor purity of the high-risk group were significantly higher than those of the low-risk group (Wilcoxon rank-sum test, *p*-value<0.001) (Supplementary Figures S1A–C), while the tumor purity was significantly lower than that in the low-risk group (Supplementary Figures S1D). We also explored the relationship of risk score with the immune score, stromal score, ESTIMATE score, and tumor purity using linear fitting. The risk score was positively correlated with the immune score (R-squared = 0.12, *p*-value = 1.155e-12), stromal score (R-squared = 0.53, *p*-value = 3.52e-69), and ESTIMATE score (R-squared = 0.35, *p*-value = 1.62e-39) (Figures 7A–C), but negatively correlated with tumor purity (R-squared = 0.36, *p*-value = 5.706e-41) (Figure 7D). The above results suggested that the higher the immune activity of patients with GC, the more likely they are to have a poor prognosis.

## Discussion

The TME plays an important role in the occurrence and development of cancer. In order to identify the prognostic signature genes associated with the TME of GC, we first identified 23 differentially expressed TME-related genes between normal samples and GC samples. Then, we further identified 3 prognostic-related genes via univariate Cox regression analysis and LASSO regression analysis. Finally, we obtained a three-gene signature (LPPR4, ADAM12, NOX4) through a risk score model constructed using multivariate Cox regression analysis in the TCGA-STAD dataset, and verified the accuracy of the model for predicting the overall survival in three independent GEO datasets. The results indicated that the risk score model could divide the patients in TCGA-STAD dataset and three GEO datasets into high-risk and low-risk groups, and the high-risk group had a poor prognosis. The time-dependent ROC curve analysis in the TCGA-STAD and GEO datasets also confirmed the accuracy and robustness of the risk score model.

In order to understand the impact of other factors on the genes we screened, we analyzed some covariates including age, gender, grade, and stage. First, we employed the multivariate logistic regression to test if the differentially expressed genes were significant after adjusting with other clinical characteristics in the training set. The results showed that each differentially expressed gene was still significant in the logistic regression analysis (*p* < 0.05, Supplementary Table S4). Then we included the clinical characteristics and five prognostic-related genes into the LASSO regression analysis, and the three signature genes were still identified (Supplementary Table S5). To further test if our risk score is an independent prognostic factor, we performed multivariate Cox proportional hazard regression to analyze the prognostic effects of the risk score and other common clinical factors, including age, grade, stage, and lymph node count, and the risk score also showed to be significant (Figure 3E). Therefore, the three-gene signature could be used as an independent prognostic factor to predict the overall survival of GC patients.

The three genes in our signature are all stromal signature genes. A number of studies have revealed that the signature genes of stromal cells and immune cells interact extensively in the TME. Fibroblasts as the major stromal cells form a protective barrier, and generally avoid tumor cells to be recognized and eliminated by the immune cells. They are further found to regulate the extracellular matrix and growth factors to promote tumor growth and metastasis (Orimo et al., 2005; Scherz-Shouval et al., 2014). Among the three identified TME-based prognostic genes (LPPR4, ADAM12, NOX4), ADAM12, which was found to be overexpressed in small cell lung cancer patients, has been proven to be a potential prognostic biomarker for cancer (Iba et al., 1999). Current research shows that NOX4 can predict the recurrence of GC patients after surgery (Lee et al., 2014). Although there is no research about LPPR4 in GC, its function in the early stage and progression of GC deserves further study.

We also performed function enrichment analysis with GSEA to explore the potential molecular mechanisms of the three signature genes. Interestingly, the results showed that the high expression of the three signature genes is all associated with the cytokine-cytokine interaction (Figures 5A–C), which was proposed to be a promising approach for diagnosis, classification, and prognosis of GC (Quan et al., 2017). To further explore the activity of immunity in TME, we evaluate the differential infiltration of immune cells between high-risk and low-risk GC patients. The most immune cells in the high-risk group showed higher levels than that of the low-risk group. Moreover, most of the HLA-related genes in the high-risk group were also highly expressed, which indicated that the local immune regulation or immune response was more active in high-risk GC patients. We finally tested the correlation of risk score with the immune score, stromal score, ESTIMATE score, and tumor purity. It was shown that the higher the risk score, the higher the immune score and stromalscore. The above results indicate that the high-risk GC patients may possess high immune activity. Overall, our study revealed that some TME cells are more active in the non-immunotherapy GC patients, and it is also consistent with the result of lower immune activity but longer survival time in the low-risk group.

After analyzing the expression of immune checkpoint genes in the TCGA-STAD data set, we observed that the expression of immune checkpoint genes in the high-risk group was higher than that in the low-risk group. It implied that the high-risk group may be more responsive to immunotherapy, but whether patients in the high-risk group are more suitable for corresponding immunotherapy than the low-risk group remains for further study.

## Data Availability

The original contributions presented in the study are included in the article/Supplementary Material, further inquiries can be directed to the corresponding authors.
