# Supplementary material for: Development and Validation of a Three-Gene Prognostic Signature Based on Tumor Microenvironment for Gastric Cancer
Source: Front Genet. 2022 Feb 1;12:801240. doi: 10.3389/fgene.2021.801240 (PMC8843853; doi:10.3389/fgene.2021.801240)
Supplement: Supplementary file 1 [file DataSheet1.PDF]

Supplementary Material

Development and validation of a three-gene prognostic signature based on tumor microenvironment for gastric cancer

Qian Wang[1,†], Xiangmei Li[1,†], Yahui Wang[1,†], Jiayue Qiu[1,†], Jiashuo Wu[1], Ying Jiang[3,*], Qingfei Kong[2,*], and Junwei Han[1,*]

Inventory of Supplementary Information

1. Supplementary Table S1-S5

2. Supplementary Figure S1

Supplementary Table 1. The datasets enrolled in the study.

Dataset	Platform	No. of patients	Median OS	Download URL
TCGA-STAD	IlluminaHiSeq 2000	407	8.5 (month)	https://xena.ucsc.edu/
GSE26901	Illumina HumanHT-12 V3.0	108	45.5 (month)	https://www.ncbi.nlm.nih.gov/geo/
GSE13861	Illumina HumanWG-6 v3.0	65	87 (month)	https://www.ncbi.nlm.nih.gov/geo/
GSE15459	Affymetrix Human Genome U133 Plus 2.0	192	18.97 (month)	https://www.ncbi.nlm.nih.gov/geo/

Supplementary Table 2. Multivariable Cox regression analysis of three signature genes in TCGA training cohort.

Gene	Coef	HR	%95 CI	P-value
LPPR4	0.206	1.228	0.933 to 1.616	0.143
ADAM12	0.146	1.157	0.839 to 1.595	0.374
NOX4	0.131	1.140	0.774 to 1.677	0.507

Coef, regression coefficient; CI, confidence interval; HR, hazard ratio.

Supplementary Table 3. Top five most significant pathways for each gene.

Gene	Pathway ID	Size	ES	p-value	FDR
LPPR4	KEGG_CYTOKINE_CYTOKINE_RECEPTOR_INTERACTION	260	0.385935	1.00E-10	2.28E-09
	KEGG_NEUROACTIVE_LIGAND_RECEPTOR_INTERACTION	271	0.482069	1.00E-10	2.28E-09
	KEGG_NEUROACTIVE_LIGAND_RECEPTOR_INTERACTION	356	0.562084	1.00E-10	2.28E-09
	KEGG_OXIDATIVE_PHOSPHORYLATION	101	-0.43935	1.00E-10	2.28E-09
	KEGG_PARKINSONS_DISEASE	99	-0.39507	1.00E-10	2.28E-09
ADAM12	KEGG_CYTOKINE_CYTOKINE_RECEPTOR_INTERACTION	260	0.448199	1.00E-10	2.63E-09
	KEGG_DRUG_METABOLISM_CYTOCHROME_P450	70	-0.50679	1.00E-10	2.63E-09
	KEGG_METABOLISM_OF_XENOBIOTICS_BY_CYTOCHROME_P450	68	-0.54205	1.00E-10	2.63E-09
	KEGG_OLFACTORY_TRANSDUCTION	356	0.655399	1.00E-10	2.63E-09
	KEGG_OXIDATIVE_PHOSPHORYLATION	101	-0.34753	1.00E-10	2.63E-09
NOX4	KEGG_CYTOKINE_CYTOKINE_RECEPTOR_INTERACTION	260	0.434682	1.00E-10	2.23E-09
	KEGG_METABOLISM_OF_XENOBIOTICS_BY_CYTOCHROME_P450	68	-0.45902	1.00E-10	2.23E-09
	KEGG_NEUROACTIVE_LIGAND_RECEPTOR_INTERACTION	271	0.415287	1.00E-10	2.23E-09
	KEGG_OLFACTORY_TRANSDUCTION	354	0.827917	1.00E-10	2.23E-09
	KEGG_OXIDATIVE_PHOSPHORYLATION	101	-0.38897	1.00E-10	2.23E-09

Size, Number of genes in the pathway; ES, Enrichment score

Supplementary Table 4. Logistic regression of differentially expressed genes and clinical characteristics.

	Estimate	95%CI L	95%CI H	P-value
**age**	**-0.067**	**-0.12**	**-0.022**	**0.0052**
gender	-0.18	-1.1	0.71	0.7
grade	0.036	-0.7	0.66	0.92
stage	0.063	-0.07	0.19	0.35
**P2RY14**	**-1.6**	**-2.1**	**-1.2**	**4.40E-11**
**age**	**-0.054**	**-0.12**	**-0.022**	**0.021**
gender	-0.32	-1.1	0.71	0.48
grade	-0.56	-0.7	0.66	0.14
stage	0.036	-0.07	0.19	0.6
**IFI30**	**5**	**-2.1**	**-1.2**	**2.70E-09**
age	-0.034	-0.12	-0.022	0.069
gender	-0.41	-1.1	0.71	0.32
grade	-0.39	-0.7	0.66	0.27
stage	0.033	-0.07	0.19	0.59
**PVRIG**	**7.1**	**-2.1**	**-1.2**	**0.00018**
age	-0.023	-0.12	-0.022	0.23
gender	-0.22	-1.1	0.71	0.62
grade	-0.6	-0.7	0.66	0.11
stage	0.037	-0.07	0.19	0.56
**SFRP4**	**0.74**	**-2.1**	**-1.2**	**2.80E-07**
**age**	**-0.043**	**-0.12**	**-0.022**	**0.037**
gender	-0.12	-1.1	0.71	0.8
grade	-0.6	-0.7	0.66	0.13
stage	0.039	-0.07	0.19	0.56
**THBS2**	**1.1**	**-2.1**	**-1.2**	**2.60E-09**
age	-0.048	-0.12	-0.022	0.11
gender	-0.51	-1.1	0.71	0.4
grade	-1.1	-0.7	0.66	0.055
stage	-0.0035	-0.07	0.19	0.97
**COL10A1**	**12**	**-2.1**	**-1.2**	**0.00013**
**age**	**-0.042**	**-0.12**	**-0.022**	**0.049**
gender	-0.3	-1.1	0.71	0.53

grade	-0.8	-0.7	0.66	0.051
stage	-0.0078	-0.07	0.19	0.91
**SULF1**	**1.2**	**-2.1**	**-1.2**	**2.90E-10**
age	-0.044	-0.12	-0.022	0.057
gender	-0.51	-1.1	0.71	0.29
**grade**	**-1.3**	**-0.7**	**0.66**	**0.0035**
stage	0.012	-0.07	0.19	0.86
**MSR1**	**2.9**	**-2.1**	**-1.2**	**1.20E-08**
**age**	**-0.052**	**-0.12**	**-0.022**	**0.03**
gender	-0.37	-1.1	0.71	0.47
grade	-0.73	-0.7	0.66	0.079
stage	0.0012	-0.07	0.19	0.99
**FAP**	**4.8**	**-2.1**	**-1.2**	**7.60E-08**
**age**	**-0.04**	**-0.12**	**-0.022**	**0.048**
gender	-0.44	-1.1	0.71	0.34
grade	-0.37	-0.7	0.66	0.32
stage	0.015	-0.07	0.19	0.82
**SCUBE2**	**-1.5**	**-2.1**	**-1.2**	**9.60E-10**
age	-0.024	-0.12	-0.022	0.2
gender	-0.19	-1.1	0.71	0.65
grade	-0.6	-0.7	0.66	0.1
stage	0.051	-0.07	0.19	0.43
**LPPR4**	**3.1**	**-2.1**	**-1.2**	**4.10E-05**
age	-0.021	-0.12	-0.022	0.27
gender	-0.15	-1.1	0.71	0.72
**grade**	**-0.73**	**-0.7**	**0.66**	**0.049**
stage	0.063	-0.07	0.19	0.31
**FMO1**	**3**	**-2.1**	**-1.2**	**8.50E-05**
age	-0.024	-0.12	-0.022	0.21
gender	-0.36	-1.1	0.71	0.4
grade	-0.43	-0.7	0.66	0.21
stage	0.045	-0.07	0.19	0.48
**COMP**	**1.6**	**-2.1**	**-1.2**	**3.00E-05**
age	-0.035	-0.12	-0.022	0.07

gender	-0.18	-1.1	0.71	0.66
grade	-0.39	-0.7	0.66	0.25
stage	0.048	-0.07	0.19	0.44
**LRRC15**	**1.4**	**-2.1**	**-1.2**	**0.00011**
**age**	**-0.039**	**-0.12**	**-0.022**	**0.054**
gender	-0.1	-1.1	0.71	0.81
grade	-0.73	-0.7	0.66	0.055
stage	0.016	-0.07	0.19	0.81
**TNFSF4**	**3.3**	**-2.1**	**-1.2**	**4.20E-07**
**age**	**-0.072**	**-0.12**	**-0.022**	**0.0092**
gender	-0.48	-1.1	0.71	0.41
grade	-0.48	-0.7	0.66	0.32
stage	0.0011	-0.07	0.19	0.99
**ADAM12**	**11**	**-2.1**	**-1.2**	**2.60E-07**
age	-0.035	-0.12	-0.022	0.081
gender	-0.29	-1.1	0.71	0.5
**grade**	**-0.75**	**-0.7**	**0.66**	**0.041**
stage	0.04	-0.07	0.19	0.54
**TFEC**	**2.8**	**-2.1**	**-1.2**	**3.40E-05**
**age**	**-0.039**	**-0.12**	**-0.022**	**0.048**
gender	-0.25	-1.1	0.71	0.57
grade	-0.031	-0.7	0.66	0.93
stage	0.055	-0.07	0.19	0.37
**HDC**	**-1.9**	**-2.1**	**-1.2**	**2.50E-08**
**age**	**-0.05**	**-0.12**	**-0.022**	**0.017**
gender	-0.37	-1.1	0.71	0.4
grade	-0.16	-0.7	0.66	0.62
stage	0.035	-0.07	0.19	0.62
**MMP3**	**1.4**	**-2.1**	**-1.2**	**2.80E-05**
**age**	**-0.04**	**-0.12**	**-0.022**	**0.057**
gender	-0.24	-1.1	0.71	0.59
grade	-0.6	-0.7	0.66	0.13
stage	0.027	-0.07	0.19	0.68
**NOX4**	**6.9**	**-2.1**	**-1.2**	**2.20E-07**
**age**	**-0.068**	**-0.12**	**-0.022**	**0.0096**

gender	-0.34	-1.1	0.71	0.55
grade	-0.2	-0.7	0.66	0.67
stage	-0.076	-0.07	0.19	0.34
**WNT2**	**9.8**	**-2.1**	**-1.2**	**2.10E-06**
**age**	**-0.047**	**-0.12**	**-0.022**	**0.03**
gender	-0.12	-1.1	0.71	0.79
**grade**	**-0.87**	**-0.7**	**0.66**	**0.032**
stage	0.059	-0.07	0.19	0.4
**CXCL9**	**1.1**	**-2.1**	**-1.2**	**7.50E-09**
**age**	**-0.042**	**-0.12**	**-0.022**	**0.042**
gender	-0.28	-1.1	0.71	0.51
grade	-0.2	-0.7	0.66	0.54
stage	0.049	-0.07	0.19	0.43
**EGFL6**	**2.1**	**-2.1**	**-1.2**	**2.40E-06**

Supplementary Table 5. LASSO regression analysis of prognostic-related genes and clinical characteristics.

	**coefficient**
**gender**	0.384
**stage**	0.085
**age**	0.024
**grade**	0.099
**COL10A1**	/
**FAP**	/
**LPPR4**	0.380
**ADAM12**	0.061
**NOX4**	0.077

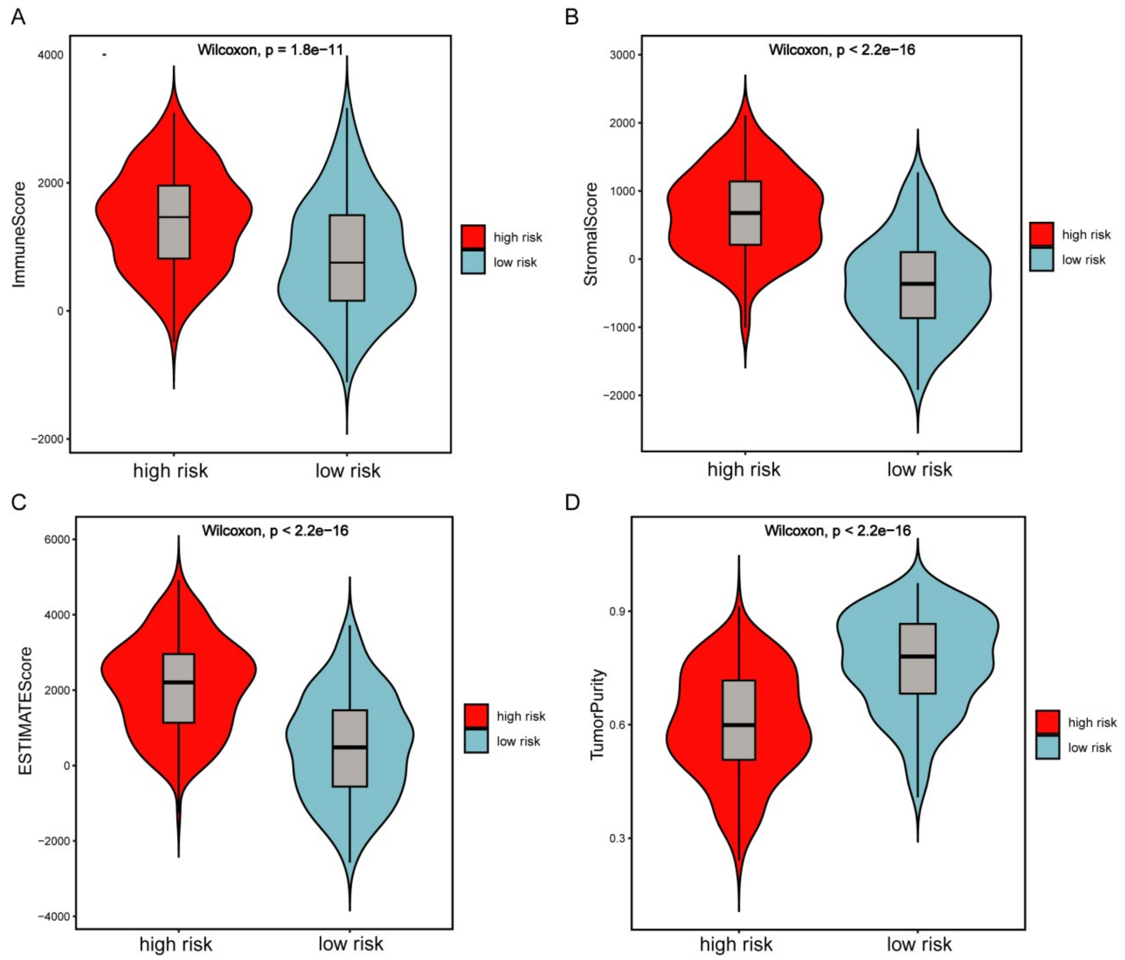

Supplementary Figure S1. Difference of the immune score, stromal score, ESTIMATE score, and tumor purity between high-risk groupand low- risk group**.** (A) Immune score. (B) Stromal score. (C) ESTIMATE score. (D) Tumor purity.
